# When It’s Good to Feel Bad: An Evolutionary Model of Guilt and Apology

**DOI:** 10.3389/frobt.2018.00009

**Published:** 2018-03-02

**Authors:** Sarita Rosenstock, Cailin O’Connor

**Affiliations:** ^1^Logic and Philosophy of Science, University of California Irvine, Irvine, CA, United States

**Keywords:** evolutionary game theory, game theory, guilt, moral emotions, prisoner’s dilemma

## Abstract

We use techniques from evolutionary game theory to analyze the conditions under which guilt can provide individual fitness benefits, and so evolve. In particular, we focus on the benefits of guilty apology. We consider models where actors err in an iterated prisoner’s dilemma and have the option to apologize. Guilt either improves the trustworthiness of apology or imposes a cost on actors who apologize. We analyze the stability and likelihood of evolution of such a “guilt-prone” strategy against cooperators, defectors, grim triggers, and individuals who offer fake apologies, but continue to defect. We find that in evolutionary models guilty apology is more likely to evolve in cases where actors interact repeatedly over long periods of time, where the costs of apology are low or moderate, and where guilt is hard to fake. Researchers interested in naturalized ethics, and emotion researchers, can employ these results to assess the plausibility of fuller accounts of the evolution of guilt.

## 1 Introduction

Some emotions provide fairly straightforward fitness advantages. Fear, for example, provides obvious evolutionary benefits, like avoiding predation by tigers. Guilt poses more of an evolutionary puzzle. Deem and Ramsey (2016b) point out that this emotion is associated with behaviors that, initially, seem maladaptive. Guilt-prone individuals behave altruistically, even in cases where they do not expect to be caught. Those who feel guilty after a transgression accept punishment readily, and even punish themselves. For this reason, one might think that guilt evolved for its benefits to human groups, rather than individuals. But the evolution of traits which benefit a group while harming individuals is notoriously fraught. Philosophers interested in the role of moral emotions as underpinnings of naturalized ethics (Joyce, 2007; Ramsey and Deem, 2015; Deem and Ramsey, 2016a,b) have recently begun to ask: how do we account for the evolution of this puzzling emotion?

Evolutionary game theory is a branch of mathematics used to model the evolution of strategic behavior in humans and animals. This framework is not traditionally employed to understand the evolution of emotions because emotions, simpliciter, are not behaviors. Extensive literature from evolutionary game theory on the evolution of cooperation, altruism, and apology can shed light on the evolution of guilt, however, by showing where and when guilt can provide individual fitness benefits to actors by dint of causing adaptive behaviors.^1^ In this paper, we discuss implications of previous results in evolutionary game theory for the evolution of guilt. We also provide novel modeling work, focusing especially on the conditions under which guilty apology can evolve. As we show, in evolutionary models where actors play an iterated prisoner’s dilemma and sometimes err, guilt can evolve to facilitate forgiveness and social reparation. Guilt functions in this setting by either ensuring the trustworthiness of apology, or by leading apologizers to pay a cost. This model adds to previous literature on the evolution of apology by showing how both emotion-reading and costly apology can interact to stabilize guilt, and by exploring in greater depth the conditions under which guilty apology is particularly likely to evolve.^2^

In Section 2, we describe the inferential strategy by which we use evolutionary game theoretic results to provide insight into the evolution of guilt. We also discuss basic insights into the conditions under which guilt provides individual fitness benefits to actors. In Section 3, we present our evolutionary model of guilty apology and clarify conditions under which guilt is likely to evolve to play this strategic role. As we will show, guilty apology is more likely to evolve when guilt is hard to fake, actors interact repeatedly over long periods of time, and the costs to apology are not too high. We argue that these models can help determine conditionals of the form “ceterus paribus, if x obtains, guilt provides greater individual fitness benefits” that emotions researchers can employ in forming and assessing more detailed accounts of the evolution of guilt. In addition, these modeling results may be informative for those working with artificial systems of interactive agents.

## 2 Previous Work: Evolutionary Game Theory and Guilt

Evolutionary game theoretic models involve two basic elements—games and dynamics. Games are simplified representations of strategic interactions. Dynamics specify how a population of actors playing a game will change, or evolve, Which behaviors (or strategies) in the game will become more prevalent as evolution progresses? Which will disappear? The answer will typically depend on how beneficial those strategies turn out to be for the actors.

Games, in evolutionary models, are characterized by three things—players, strategies, and payoffs.^3^ These correspond to the agents involved in an interaction, their possible behaviors, and what they get for their behaviors, respectively. There is no resource here for representing the emotional state of an actor. This might make the evolutionary game theoretic framework seem like a poor one for elucidating the evolution of guilt. Inasmuch as emotions in humans are causally connected to behaviors, however, we can use these models to gain insight into what functional role emotions might play. If we see a behavior *X* selected for in environment *Y*, and we know that emotion *Z* causes that behavior in humans, we can conclude that emotion *Z* should come under positive selection pressure in *Y* as well (O’Connor, 2016).

Guilt, our focus here, is associated with three classes of behaviors in humans. First, the anticipation of guilt prevents social transgression (Tangney et al., 1996). It is thus correlated with altruistic and cooperative behavior in humans, as well as decreased norm violation (Regan, 1971; Ketelaar and Au, 2003; Malti and Krettenauer, 2013). Second, the experience of guilt leads to a suite of reparative behaviors including apology, gift giving, acceptance of punishment, and self-punishment (Silfver, 2007; Nelissen and Zeelenberg, 2009; Ohtsubo and Watanabe, 2009). Lastly, expressions of guilt lead to decreased punishing behaviors, and forgiveness, by group members (Eisenberg et al., 1997; Gold and Weiner, 2000; Fischbacher and Utikal, 2013). If we find, in evolutionary models, that these sorts of *behaviors* provide selective advantages to individuals, we identify a situation in which guilt can provide a selective advantage as well. O’Connor (2016) identifies three sets of evolutionary game theoretic results that can inform the evolution of guilt. Before continuing to our work on guilty apology, we will give a quick overview of these results.

### 2.1 Guilt Leads to Punishment Avoidance

This result employs the famous prisoner’s dilemma game, which we will describe at length in Section 3, to model the evolution of altruism. In evolutionary game theory, *altruism* refers to any behavior in which an agent decreases their own payoff in order to increase another agent’s payoff. The primary mechanisms that can create individual level benefits for altruism are *reciprocity* and *punishment*.^4^

If actors can remember others’ past actions and *reciprocate*—by behaving altruistically toward altruists and selfishly toward the selfish—altruism can be directly beneficial to the individual. Emotions that promote altruism, such as guilt, are likewise beneficial in such a scenario. Guilt can provide a benefit to an agent who, anticipating feeling badly about taking advantage of another group member, elects not to, and thus escapes reciprocal selfishness from that group member.

When actors *punish* those who fail to behave altruistically, likewise altruism, and guilt, are directly beneficial to the individual.^5^ If an actor anticipates the experience of guilt, and so chooses not to steal from a friend, say, this might be beneficial if that actor then escapes punishment for their behavior. Human groups engage both in reciprocity and in punishment, suggesting that guilt can provide a selective advantage in these groups by preventing failures of altruism (Boyd et al., 2003; Boyd and Richerson, 2009).

### 2.2 Guilt Stabilizes Risky Cooperation

In models that employ the “stag hunt” game to represent mutually beneficial, but risky, cooperation, guilt can benefit actors by stabilizing such cooperative behavior. In these types of interactions, it always benefits actors to cooperate when their partners do as well, even in the face of transient temptation to do otherwise. An emotion, like guilt, that promotes cooperation will then provide individual benefits to any actor in a generally cooperative group of this sort. For example, suppose two actors have agreed to hunt a stag together, or cooperate, but one is tempted to hunt a hare instead, i.e., to seek short term, less risky, payoff. If anticipation of guilt keeps this actor focused on the stag hunt, and their partner pulls through, they will eventually receive greater rewards for sticking to the larger, if riskier, joint project. Alexander (2007) shows that cooperation in the stag hunt is especially likely to evolve in groups where the same actors tend to keep interacting, as in early human groups. (See Skyrms (2004) for more on the stag hunt and the evolution of cooperation.) To be completely explicit, the benefit guilt provides here is in keeping actors dedicated to risky joint projects.

### 2.3 Guilt Allows Agents to Recover from Mistakes

Apology can benefit individuals playing the iterated prisoner’s dilemma—a version of the game where the same actors repeatedly are engaged in an opportunity for altruism. Strategies that reciprocate by refusing to behave altruistically toward selfish types can do well, but they suffer a problem when faced with accidental bad behavior by a partner. These strategies can become locked in a spiral of mutual negative reciprocation, which hurts all involved.

Imagine, for example, interactive partners who regularly share meat. Suppose that after one hunt a partner fails to do so because they are especially hungry. If these actors reciprocate, the slighted partner will fail to share meat after the next hunt, leading the other partner to fail to share in following interactions, and so on. Actors who apologize, and accept the apologies of group members, can gain thus an advantage in such conditions.^6^ These apologies can work if they are costly (Okamoto and Matsumura, 2000; Ohtsubo and Watanabe, 2009; Ho, 2012; Han et al., 2013; Martinez-Vaquero et al., 2015; Lenaerts et al., 2017), if they are unfakeable, or if they combine elements of costly and unfakeable apology (O’Connor, 2016). (In the next section, we will explain at length why this is so.) Pereira et al. (2016) connect these models of apology to the evolution of guilt. Pereira et al. (2017a,b) follow another tack and present an evolutionary model where guilt causes self-punishment in the presence of a guilt-prone interactive partner. These results indicate that the often costly apologies generated by the experience of guilt may nonetheless provide individual fitness benefits in the long run by incentivizing group members to accept guilty actors into the social fold after bad behavior.

The models we present here expand on this body of work. Our paper explores the issue from a different angle by focusing on the trade-off between costs of apology and unfakeability in stabilizing the evolution of guilty apology. This focus on emotion-reading and unfakeable signals means that our model is more tuned into explaining guilt in particular—which as an emotion acts as its own signal between humans—rather than apology in general. In addition, the results here provide a robustness check on some of these previous results by deriving them under substantially different modeling choices. Our model, in particular, makes fewer assumptions about the rationality of players, and thus is again more directed at the particular case of guilt.^7^

Note that while all three sets of results mentioned describe conditions under which guilt can be selective, there is nothing here suggesting that guilt is somehow necessary in these situations. An organism does not need to feel guilt to behave altruistically. This may lead one to ask: how do these models tell us anything about guilt, rather than simply altruistic, cooperative, and apologetic behavior? Of course, evolution can solve problems in different ways. Observing that giraffes evolved long necks to reach trees does not mean that giraffes had to evolve long necks to eat. The fact that these human behaviors might be caused by other cognitive mechanisms, though, is besides the point in this case. We know that guilt *did* evolve, so the explanatory demand is to say what conditions might have caused this despite the possible fitness detriments associated with guilt, not to show that guilt is the only way to solidify altruism, cooperation, and apology in humans.

## 3 Guilty Apology

We now turn to our model of guilty apology.

A *prisoner’s dilemma* is a two-player game in which each player has two possible strategies: “cooperate” and “defect”. If both players cooperate, they both get a moderate payoff (2, in our model). If one cooperates and one defects, the cooperator gets nothing (0) and the defector gets a large payoff (3). If they both defect, they both get a small payoff (1).^8^ In other words, mutual cooperation is preferable to mutual defection, but each player does best to defect regardless of the other player’s choice. Cooperation in this game is an altruistic strategy, as players who choose it incur a cost and increase their partner’s payoff. It should not be confused with cooperation as modeled in the stag hunt, where actors obtain mutual benefits.

**Figure 1:**
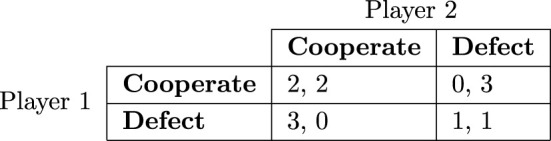
A payoff table of the prisoner’s dilemma. Strategies for player one are represented by rows. Strategies for player two are represented by columns. Entries to the table show payoffs for each combination of strategies with player one listed first.

Figure 1 shows the payoff table for this game.^9^ Table entries show payoffs for each combination of strategies with player one listed first. This game is a dilemma because both players are expected to defect, despite the fact that mutual cooperation is preferred by everyone. The strategy pair where both players defect is the only set of strategies for which no player can benefit by switching, or the only *Nash equilibrium* of the game.

In an *iterated* prisoner’s dilemma, agents repeat the prisoner’s dilemma round after round with some probability *n*. One can equivalently think of as encoding the average number of rounds each encounter is expected to last, as this is given by $\frac{1}{1-n}$. For example, if n = 0.95, there will be an average of 20 rounds played. These repeated interactions allow cooperation to gain a foothold in the game via reciprocation. One such reciprocating strategy is called the “grim trigger”—players begin by cooperating, but if their partner defects they immediately switch to defection for the rest of the interaction. In this way, they cooperate with cooperators and defect with defectors, gaining the benefits from mutual cooperation and mitigating harm from defectors.

This strategy runs into problems when players have a chance of accidentally performing the wrong action—defecting instead of cooperating, or vice versa. These sorts of accidents occur for many reasons in the real world. Actors under difficult conditions may behave antisocially despite general prosocial tendencies. Actors might forget a cooperative agreement. Or actors might simply not feel cooperative today. Accidental defection causes grim triggers to permanently defect on good, mostly cooperative partners. Mutual negative reciprocation of this sort is mutually damaging.^10^ In such an environment, an apologetic strategy, which we call the “guilt-prone grim trigger”, or just guilt-prone, for short, can outperform a punitive one. Guilt-prone players act as grim triggers, but apologize after accidental defection. Upon receipt of such an apology, they forgive and forget, and so return to cooperating. Note that guilt-prone, in this context, is a behavioral strategy, rather than an explicit representation of actors who, in fact, experience some emotion. The idea is that the behavioral strategy corresponds to the actions that someone experiencing guilt would take, and so investigating the success of this strategy can tell us something about the circumstances under which guilt is adaptive.

This strategy assumes individuals combine two aspects of behavior that need not go together—apologizing after defection and forgiving those who apologize. As we will see, this can be a successful strategy, but its success depends on the presence of both behaviors. In this way, it mimics “secret handshakes”—where two actors use some private signal to determine who to cooperate with, and only trust those who know the signal (Robson, 1990). It is also similar to green beards in the biological realm—traits that need not have anything to do with altruism, but that are used to coordinate altruism among those with the trait (Gardner and West, 2010). One missing part of the account here is how these behaviors become linked in the first place. This lack, however, does not undermine the account we will provide of the stability and evolution of a strategy in which they are linked.

One problem with the guilt-prone strategy is that an apology will not effectively signal guilt if defectors can also use it to convince their partners to cooperate, even though they intend to continue to defect. Another way of putting this is that guilty apology might not be evolutionarily viable if fake apologizers, which we will call fakers, can take advantage of the forgiving nature of guilt-prone players (Martinez-Vaquero et al., 2015).

As we mentioned in the last section, there are two lines of defense against such fakers. One is for guilty apologies to be unfakeable. This relates to arguments by Frank (1988) that moral emotions, such as guilt, evolve as honest signals of cooperative intent in humans. Empirical evidence suggests that humans do trust signals of guilt from group members to some degree when deciding whether to forgive and forget, but that guilt, unlike some emotions, is not associated with stereotyped facial and body postures (Deem and Ramsey, 2016b). In other words, it is not entirely unfakeable. For this reason, in our model, we assume that guilt-prone types always manage to successfully apologize and fakers are successful with some probability less than or equal to one but greater than zero.

Another way to discourage fakers is to impose a cost for apologizing. When guilt-prone types apologize to each other, they are able to re-enter a potentially long cooperative engagement where they both reap the benefits of mutual aid. This means that the expected benefit of apologizing is high. When fakers apologize, they defect the next round, necessitating another costly apology if they wish to re-enter the social fold. This means that the benefit to fakers of apologizing is a short period of defection, which yields a relatively small payoff. These differential benefits mean that paying an identical cost will be less worthwhile for fakers than guilt-prone types under many conditions. For example, imagine two actors, each of whom has stolen a cupcake and is deciding whether to issue a costly apology. The actor who is planning a long, cooperative life with the cupcake maker will receive a large benefit from doing so. The actor who will steal a cupcake tomorrow only receives a small benefit before having to pay the cost again.

To summarize, our model works as follows. We assume that a population of actors plays the iterated prisoner’s dilemma where every round the game continues with probability *n*. Each round, there is a probability *a* that actors accidentally perform the wrong action—that those who usually cooperate in fact defect, or vice versa. (This is the condition under which reciprocation can be harmful, and apology is potentially useful.) The strategies in the population are:

C—Unconditional cooperation, or always cooperate in every round
D—Unconditional defection, or always defect in every round
GT—Grim trigger, cooperate unless your partner defects, and then defect for every following round
GP—Guilt-prone, play grim trigger, apologize upon defection, and cooperate with those who apologize
F—Faker, always defect, apologize upon defecting

There is some probability *p* ≤ *1* that fakers successfully signal guilt. And in order to apologize, actors pay a cost, as described above. To allow for the further possibility that actually guilty types pay a lower cost than fakers to convince others of their guilt we define *c* ≥ *d* where *c* is the cost of apology for guilt-prone types and *d* for fakers. The idea here is that if those who experience the real emotion make very convincing apologies, their interactive partners might not require costly reparations from them. After choosing values for our parameters, we can generate a payoff table for each strategy based on the expected outcome for playing an iterated prisoners dilemma under these conditions. For the details of these calculations, see Appendix A.

The guilt-prone strategy in this model matches empirical observations of guilt after transgression in the following ways. Guilt-prone actors are more likely to apologize. They are willing to pay a cost to do so. Upon receipt of this apology, group members decrease their punishing behavior. And guilt-prone individuals are in fact likely to behave prosocially in the future. Furthermore, guilt-prone individuals may be better at convincing others that they really do intend to cooperate in the future than those who apologize without feeling guilt. By looking at the evolution of this strategy in the model, then, we hope to gain insight into the actual conditions under which guilty apology evolves.

### 3.1 When Can Guilt Evolve?

In this section, we will address the conditions under which guilt-prone is an evolutionarily stable strategy (ESS). ESSes are strategies where populations playing them cannot be invaded by a small number of actors using a different strategy. This is because ESSes are strategies that do better against themselves than other strategies do against them.^11^

It is also the case that for the replicator dynamics, the most commonly used model of evolutionary change in evolution game theory, ESSes are stable.^12^ If populations evolve to them, they stay there, absent other forces. For this reason, an ESS analysis is a way of identifying strategies that have the potential to evolve in a model. Because of this potential, ESS analysis has been employed extensively in biology and the social sciences to gain insight into evolutionary properties of many sorts of populations.^13^^,^^14^

In the models we consider, unconditional cooperation is never an ESS, because defection does better against it than it does against itself. Unconditional defection is always an ESS, because it always does better against itself than any other strategy does against it. The grim trigger is also an ESS, for the same reason. (This is true as long as the cost for apology is not 0, in which case guilt-proneness can invade). Guilt-proneness is sometimes an ESS, because it too does better against itself than other strategies, though it is destabilized by successful fakers under some parameter values. (Fakers, in turn, are eventually replaced by defectors who do not pay costs to apologize—faking is never an ESS.^15^) As it turns out, the guilt-prone strategy is evolutionarily stable against fakers in a sizable portion of the parameter space. This means that there are many conditions under which guilt-proneness can potentially evolve. As we will see, both higher cost of apology, *c*, and lower probability of fake apologies working, *p*, helps protect guilt-prone players against fakers. For now, we will assume that *c* = *d*, or that both types pay the same cost for apology.

**Figure 2:**
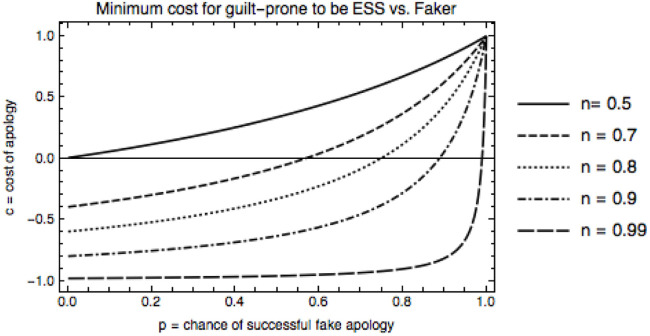
Minimum cost, *c*, for guilt-prone to be an ESS vs. faker, for each fakeability value *p*, with error rate *a* = 0.01. When faking is easier, as *p* goes to one, the cost for guilt-prone to be an ESS is higher. As *n*, increases, this cost is lower, because guilt-prone types do well in longer repeated interactions.

In order for the guilt-prone strategy to be an ESS given a fixed error rate, *a* = 0.01, and chance of repeat encounter, *n*, Figure 2 shows that the harder an apology is to fake, the cheaper the cost of apology needs to be. Alternatively, the higher the cost, the less fakeable an apology needs to be. Note that this figure only shows conditions under which guilt is stable against fakers—we will discuss other strategies shortly.

This graph also indicates that for longer interactions (larger *n*) guilt is stable under wider conditions, i.e., with lower cost, *c*, and higher fakeability, *p*. This makes sense, since the more rounds that are played on average, the more likely a guilt-prone player is to reap benefits of long interactions with other guilt-prone types, and the more likely they catch on and disbelieve a fake apology, thus depriving the faker of the benefits of defecting against a cooperator for the rest of the encounter.

**Figure 3:**
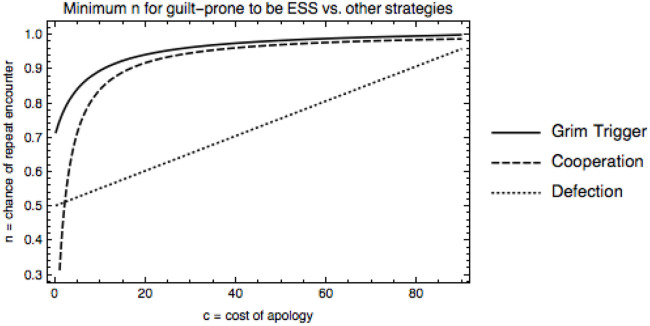
Minimum likelihood of repeated encounter, *n*, for guilt-prone to be an ESS against defectors, cooperators, and grim triggers, error rate *a* = 0.01. As the costs of apologizing increase, longer interactions are necessary for guilt-prone to be an ESS against all strategies.

One might also be interested in determining under which conditions the guilt-prone strategy is an ESS versus other strategies. When not playing against fakers, fakeability (*p*) no longer matters. Figure 3 shows the minimum length of interaction (*n*) for which the guilt-prone strategy is an ESS versus grim trigger, unconditional cooperation, and unconditional defection when the error rate is *a* = 0.01. The guilt-prone strategy is an ESS for most of the parameter space we’ve been looking at, i.e., low costs and high chance of repetition. As error rate, *a*, increases, length of play needs to increase for the guilt-prone strategy to be an ESS, but little changes in the range that we focus on. Note that in early human groups, we expect the length of repeated interaction to have been very high, meaning that guilt-prone should do well under these conditions.

Before continuing, it will be useful to take a moment to discuss the results just presented. The take-away from these models is an understanding of the conditions under which guilt, for the purposes of promoting costly apology, can potentially evolve. This model is best understood not as a “how-possibly” or just-so story for the evolution of guilt, but rather as a tool for researchers who do more detailed work on this evolution to evaluate the plausibility of various evolutionary pathways to modern, human guilt. It yields for us conditionals along the lines of “if X, then guilt provides individual benefits/comes under positive selection pressure/will evolve ceterus paribus”, that can be taken as evidence used to support or deny proper accounts of the evolution of guilt.

We can summarize these conditionals as follows: (1) When humans interact for long periods of time, guilty apology is more evolvable, because in these cases there are long, fruitful interactions to be gained by those who can forgive and forget. (2) When guilt is hard to fake, perhaps because humans are good at reading the emotions of other humans, again it has more potential to evolve. (In the same cases, note, there might be selection pressure to read these very emotions, if actors who can do so have a route to successful apology.) But actors need not be perfect emotion readers for guilt to be successful at promoting apology. (3) For various levels of fakeability, there will be costs to apology that allow guilt to evolve. In other words, if guilt creates some individual costs to the agent, which, as discussed, occurs in real human populations in the form of self-punishment, acceptance of punishment from others, and reparations, this can help guilt evolve even in populations with fakers. As described, guilt-prone types reap a disproportionately high benefit from apologizing, which makes the costs more worth their while than for fakers.

In the next section, we will expand this analysis by looking, in detail, not just at whether guilt *can* evolve to promote apology under certain conditions, but how likely this evolution is.

### 3.2 The Robustness of Guilty Apology

We have now seen that guilt-proneness can evolve in order to promote costly and/or honest apology. In this section, we will describe, in some greater detail, the conditions under which guilt is *likely* to evolve for this function. ESS analyses are useful because they tell us something about which strategies have the potential to evolve. Some ESSes, however, have very small *basins of attraction* under the replicator dynamics. A basin of attraction, for an equilibrium, is the set of population states that evolve to that equilibrium.

What does this mean? The states of an evolutionary population correspond to the possible proportions of strategies in that population—50% fakers, 10% guilt-prone, and 40% cooperators, for example, or 100% grim triggers. In an evolutionary model using the replicator dynamics, each such state will evolve based on which strategies are doing relatively well. Successful strategies will propagate, while less successful ones decline. Eventually this process will (usually) lead the population to an equilibrium, or a state where it does not evolve anymore.^16^ In our model, these equilibria are the ESSes of the game. Basins of attraction tell us what proportion of states eventually end up at each ESS. For this reason, the size of a basin of attraction tells us something about the evolvability of a strategy. Equilibrium strategies with large basins are more likely to evolve in a population than ones with small basins (assuming no knowledge about the starting state of the population).

Mutations, or noise, in evolutionary processes, which tend to occur regularly in the real world, can also move populations from one equilibrium to another. Equilibria with large basins of attraction tend to be harder to disrupt, while those with small ones are typically easier to move away from. In models explicitly representing this sort of noise, populations tend to spend most of their time at equilibria with large basins of attraction. Again, this means we should think of ESSes with large basins of attraction as likelier to evolve.

We thus want to ask: under what conditions does guilt-proneness, as represented in our model, have a larger basin of attraction? What are the factors that make it likely to evolve and be stable for the purpose of promoting apology? There are a few parameters to consider in answering this question. We can ask what happens to the basin of attraction for guilt-proneness when we vary *p*, the probability that fakers successfully trick others into trusting their apologies, *c*, the cost of apology for guilt-prone players, and *d*, the cost of apology for fakers.^17^

Let us start with *p*. In models without fakers, guilt-prone types do better. Fakers can be thought of as siphoning away the benefits of guilty apology. For this reason, holding other conditions fixed, guilt-proneness has a larger basin of attraction whenever *p* is smaller. If guilt is hard to fake, it is more likely to evolve.

The role of *c* and *d*, the costs for apology, are a little more subtle. First, consider the case where *c* = *d*, or fakers and guilt-prone types pay the same cost. When *p* is low, guilt-prone types do well against fakers. For this reason, increasing costs actually makes guilt-proneness less likely to evolve. It simply decreases the payoffs to guilt-prone types, while failing to significantly help them differentiate themselves from fakers. When *p* is higher, cost can help guilt-prone types evolve. It allows them to prove their cooperative intent compared to faker types.

**Figure 4:**
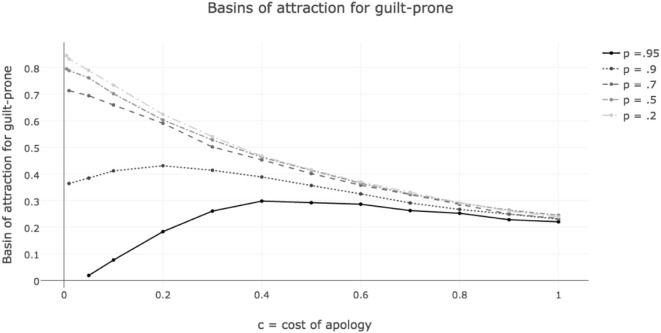
Sizes of basin of attraction for guilt-prone strategy as *c* = *d*, cost of apology for guilt-prone and fakers, and *p*, likelihood that fakers successfully apologize, vary. For high values of *p*, costs increase the basins of attraction for guilt-prone to a point. For low values of *p*, costs only hurt the evolvability of guilt-prone. Lower *p* always makes the basin of attraction of guilt-prone larger.

Figure 4 shows the sizes of the basin of attraction for guilt-proneness, as opposed to defection, in games where *p* and *c* vary, *a* = 0.01 and *n* = 0.95. The basins of attraction were measured using the discrete time replicator dynamics. Results are from 10,000 simulations run until the population was clearly converging to one of the two rest points—all play guilt-prone, or all play defect.^18^ The strategies included were unconditional cooperation (C), unconditional defection (D), guilt-prone grim trigger (GP), and faker (F). The x-axis tracks cost, *c* = *d*, which ranges from.005 to 1. The y-axis shows the basin of attraction for guilt-proneness. For *p* = 0.95, when fakers are able to almost always convince others of their cooperative intent, the optimal cost for the evolution of guilty apology, of those explored, is 0.4. For *p* = 0.9, when fakers are slightly less successful, the optimal cost is 0.2. For the smaller values of *p*, costs make guilt less likely to evolve. In other words, the easier it is for fakers to convince others they are telling the truth, the higher the costs that make guilt-prone most likely to evolve.

**Figure 5:**
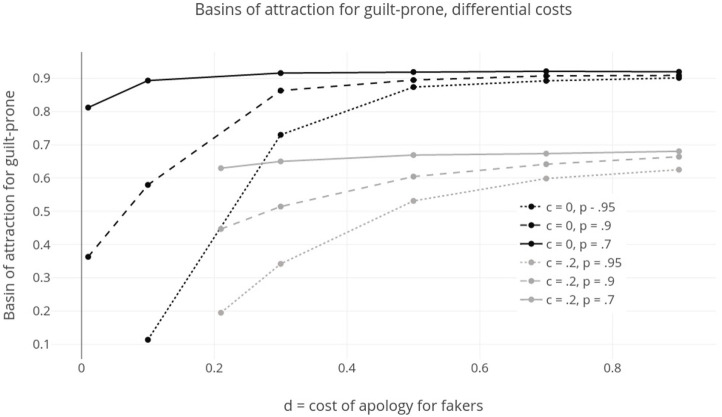
Sizes of basin of attraction for guilt-prone where *d* ≥ *c*, or costs to fakers are higher than costs to guilt-prone to apologize. When *p*, probability of faking, is lower, or when *c*, cost for apology, is smaller, or when *d*, cost for fake apology, is larger, guilt-prone has a larger basin of attraction.

The other situation worth considering here is the one where *d* > *c*, or where fakers must pay some greater cost to apologize. The idea is that their apologies are less convincing and so social partners exact an extra cost before trusting their apologies. Figure 5 shows basins of attraction for guilt in these models with probability of successful faking (*p*) held fixed at 0.95 and error rate, *a* = 0.01. Two data sets are pictured here. For the first, *c* = *0*—guilt-prone types pay no cost to apologize—and *d* ranges from 0.01 to 0.9, meaning fakers pay various costs to apologize. For the second, *c* = 0.2—a small cost for guilty apology—and *d* ranges from 0.21 to 0.9. In both cases, increasing *d*, the cost to fakers, while holding *c* fixed, increases the likelihood that guilt evolves. When there is a cost for guilt, this generally decreases the likelihood it will evolve. Both these results should be unsurprising. Costs for fakers make faking a less successful strategy, and stabilize guilt. Costs for guilty apology make guilty types less successful and allow defection to evolve more often.

In all the results just shown, we hold *a*, error rate, and *n*, probability of repeated interaction, fixed. For larger *n*, generally, guilt-proneness will be more evolvable. As long as *a* is relatively small, changes to this parameter value do not significantly alter results. As mentioned, the grim trigger is also an ESS of this model, and adding this strategy can shift evolutionary outcomes. When costs of apology are low, the presence of the grim trigger increases the basin of attraction for guilt-proneness because it disproportionately hurts defectors and fakers. When costs are higher, the grim trigger is so successful itself that it decreases the basin of attraction for guilty apology. In general, the addition of this strategy to the mix supports the claim that guilty apology is only evolutionarily viable when costs for apology are small and emotion-reading helps deter fakers.

Again, these models generate a set of conditionals that now tell us something like, “if X, then guilt-prone will be more *likely* to evolve, and to be stable, for the purposes of apology”. When guilt is easier to honestly convey, it is more likely that guilty apology will evolve and be stable. As in the last section, this makes sense. Guilt-proneness helps both players in the case of apology, one to identify a good cooperative partner even in the face of defection, and the other to convince group members to accept them back into the fold after messing up. As long as fakers can be kept at bay, this benefit obtains, and reading emotions helps this happen. Extra costs to fakers to apologize help guilty apology evolve, for the same reasons. These could obtain if, for example, group members are somewhat able to read emotions, and level extra costs for apology on those who do not seem genuine enough.

And lastly, costs for apologizing have a less straightforward impact on the likelihood that guilty apology evolves. They improve its chances when there are tricky fakers about by disincentivizing fake apology. On the other hand, they make it harder to sustain guilt because the costs straightforwardly harm the guilty individuals. The best conditions for the evolution of guilty apology would be those where actors can simply tell who is genuine and who faking. Of course, we should be so lucky.

## 4 Conclusion

Results from evolutionary models indicate that there are many conditions that can make guilt-proneness individually beneficial for actors. When it comes to benefits to guilt before bad behavior, these include the presence of reciprocating, or punishing group members, and the presence of established, mutually beneficial patterns of cooperation. When it comes to benefits after bad behavior, guilt can help actors if it allows for unfakeable apology, costly apology, or some combination of the two of these. Guilt is particularly likely to evolve and be stable for this function if it is harder to fake, either in the sense that group members do not believe fake apologizers, or in the sense that they levy higher costs to ensure the apologies of faker types. It is also especially beneficial in repeated interactions. Costs for apology improve the evolvability of guilt when fakers are more successful, but hamper the success of guilt-prone types otherwise.

One might object that the models presented here do not explicitly represent the role of culture in guilt. Culture seems likely to have played a role in the evolution of guilt and clearly plays a role in the production of guilt in modern societies. We do not mean to downplay the importance of cultural elements in the evolution of guilt. Rather, we think these models provide insight whether or not guilt, and the environment that it evolved in, are culturally evolved. To put it another way, if we, as suggested, think of these models as giving us conditions under which guilt provides significant benefits, and so is more evolvable, these conditions may be produced by a culturally evolved social environment or a more straightforwardly biologically evolved one, and furthermore, they will provide benefits for culturally produced guilty behaviors as well as biological ones. Our if-then statements are broadly applicable. This is, of course, especially useful given that the details of the evolutionary environment of humans are sometimes murky. The mathematical models presented here are one more tool to use to gain clarity.

In addition, there is interest in the AI and computation communities in understanding the role something like guilt might play in artificial systems (Pereira et al., 2016, 2017a,b). In this case, the goal of an evolutionary model of guilt is not to accurately represent the historical pathways by which guilt might have evolved, but to show possibilities for how to stabilize guilt and cooperation in an evolving system. These models elucidate such pathways.

## Author Contributions

SR was responsible for the primary mathematical work and generated all results presented in the appendix. She consulted on the writing of the MS and produced figures. CO designed the main model, performed simulation work, wrote the majority of the MS, and produced figures.

## Conflict of Interest Statement

The authors declare that the research was conducted in the absence of any commercial or financial relationships that could be construed as a potential conflict of interest.

## Footnotes

^1^For work doing just this see Han et al. (2013), Martinez-Vaquero et al. (2015), O’Connor (2016), Pereira et al. (2016, 2017a), and Lenaerts et al. (2017).

^2^Relatedly, Huttegger et al. (2015) show how in the general case of costly signaling, if signals are hard to fake the costs necessary to guarantee them are lower.

^3^Games can also represent information that agents have about each other and the structure of the game, but in evolutionary scenarios, this element is less important.

^4^See Nowak (2006) for an overview of the evolution of altruism in the prisoner’s dilemma. Other factors like group and kin selection can lead to the evolution of altruism, but not through individual fitness benefits. Network reciprocity, where individuals encounter and copy neighbors, can also stabilize altruism, but in each of these cases any individual who cooperates would do better to switch to defecting. For this reason, in thinking about when guilt provides a selective advantage we focus on situations where it will be individually beneficial. For theoretical discussion of group and kin selection explanations of guilt, see Deem and Ramsey (2016b).

^5^The evolution of punishment presents the so-called “second order free rider problem.” If punishment is costly, individuals should prefer to leave punishment to others, and systems of punishment should dissolve. While models have been developed to solve this problem, for our purposes the observation that really matters is that for whatever reason humans do, in fact, punish transgressive group members. Panchanathan and Boyd (2004) give a good overview of this literature and show how indirect reciprocity can circumvent the second-order free rider problem.

^6^This is not to say that apology is the only solution to this sort of retribution problem. The strategy “tit-for-two-tats” does not begin retaliating until a partner has defected twice, and so is less likely to enter spirals of defection (Axelrod and Axelrod, 1984). Likewise, “generous tit-for-tat,” where actors usually reciprocate, but sometimes play cooperation in response to defection instead, can invade a population playing tit-for-tat, and maintain a lower level of retribution (Nowak and Sigmund, 1992). The Pavlov strategy involves players who cooperate whenever their last play matched that of their opponent and solves the retribution problem (though it is vulnerable to defectors) (Nowak and Sigmund, 1993). The goal here is not to show that apology is the only solution to spirals of defection, but simply that it is a solution, and to argue that this can help explain the evolution of guilty apology. (Note also that since guilt plays a role in other prosocial behaviors, it is available for co-option to solve the retribution problem.)

^7^For example, in showing how costs can stabilize apology in the iterated prisoner’s dilemma, Martinez-Vaquero et al. (2015) focus on the role of commitments to cooperate between agents who must then pay a cost upon breaking these commitments.

^8^We choose these values somewhat arbitrarily rather than looking at a generalized version of a prisoner’s dilemma. While variation to the payoffs may lead to some small differences in our analyses (for example, sizes of basins of attraction), it will not lead to qualitative differences. Thus, for tractability purposes, we follow many past actors in simply choosing these payoffs.

^9^Note that there is nothing special about the particular numbers in this payoff table. This game is a prisoners’ dilemma by virtue of the ordering of the payoffs only. We fixed these values for computational expedience, but changing them does not effect the qualitative features of the results we will present.

^10^O’Connor (2016) also looks at “tit-for-tat”, another reciprocating strategy, as do Martinez-Vaquero et al. (2015) and find similar results to ours. We focus on the grim trigger here because while the qualitative results are similar between the two models, the analysis is neater for grim trigger.

^11^To be more precise strategy *x_i_* is an ESS if *u*(*x_i_*,*x_j_*) is the payoff of strategy *x_i_* played against *x_j_* and: (1) *u*(*x_i_*, *x_i_*) > *u*(*x_i_*, *x_j_*) or (2) *u*(*x_i_*, *x_i_*) = *u*(*x_i_*, *x_j_*) and *u*(*x_i_*, *x_j_*) > *u*(*x_j_*, *x_j_*) for all *x_j_* ≠ *x_i_*.

^12^For more on the replicator dynamics, including the discrete-time version employed in the next section, see Weibull (1997).

^13^See Smith and Price (1973) for more on the role of ESS analysis in evolutionary game theory.

^14^All this said, ESSes are not the only stable states under the replicator dynamics, and sometimes ESSes are quite unlikely to evolve. Huttegger and Zollman (2013) discuss problems with ESS methodology as opposed to dynamical analyses. O’Connor (2017) discusses a particular example, the evolution of learning, where ESS analysis is misleading. In the next section, we will move to a dynamical analysis for these reasons.

^15^This is the case unless the costs to apologizing are zero, in which case defection cannot invade faking as they both simply defect against each other and pay no costs to do so.

^16^See O’Connor (2016) for a bit more on the replicator dynamics in this sort of model.

^17^We measure basins of attraction by randomly initializing 10k populations for each parameter value and using the discrete time replicator dynamics to determine the equilibrium each starting point ends up at. For more on this concept, see, for example, Sandholm (2010).

^18^Simulation starting points were chosen over all possible population proportions using a uniform distribution.

## Appendix

### A. Calculations of Expected Return

Here, we show how we calculated the payoffs used in the above analysis for the iterated prisoner’s dilemma.

The payoff table in Figure 1 gives the expected return for a single round based on each of four possible action pairs performed by the players. However, this does not take in to account the fact that both players have a chance *a* of performing the wrong action, and that guilt-prone players and fakers pay a cost *c* when they defect, which changes their expected return from defecting. Moreover, we’re primarily interested in total expected return over some unknown number of repeated interactions as determined by *n*. For some strategies, the action performed in a given round depends on actions of the other player in previous rounds: whether they made mistakes, and (for guilt-prone players vs. fakers) whether they effectively apologized for defecting.

We’ll first calculate expected single-round payoffs for each intended action pair, and then use this information to calculate total expected return for a complete interaction between each pair of strategies. This total does not denote actual return from an interaction, as interactions involve probabilistic elements in the form of *n*, *a*, and *p*, but can be thought of as an average over a large number of such interactions.

#### Expected Return Each Round

Here, we show how to calculate each single-round action’s expected return, by taking the sum of entry in the payoff table for player 1 multiplied by the probability that they get that payoff when performing that action, considering player 2’s action, the error rate *a*, and the cost of apologizing *c* (if the strategy involves apology).

*CC* denotes the expected return for each player for a round in which both players intend to cooperate. This is given by:

$$\mathrm{CC}=2{\left(1-a\right)}^{2}+3a\left(1-a\right)+0\left(1-a\right)a+1a^{2}=2-a$$

*CC**: expected return for a player who intends to cooperate this round, but will pay a cost of *c* to apologize if they accidentally defect, playing another player who intends to cooperate.

$$CC^{*}=2{\left(1-a\right)}^{2}+\left(3-c\right)a\left(1-a\right)+0\left(1-a\right)a+\left(1-c\right)a^{2}=2-a-\mathrm{ca}$$

*CD*: expected return for a player who intends to cooperate this round, playing a player who intends to defect.

$$\mathrm{CD}=0{\left(1-a\right)}^{2}+1a\left(1-a\right)+2\left(1-a\right)a+3a^{2}=3a$$

*CD**: expected return for a player who intends to cooperate this round, but will pay a cost of *c* to apologize if they accidentally defect, playing a player who intends to defect.

$$CD^{*}=0{\left(1-a\right)}^{2}+\left(1-c\right)a\left(1-a\right)+2\left(1-a\right)a+\left(3-c\right)a^{2}=3a-\mathrm{ca}$$

*DC*: expected return for a player who intends to defect this round, playing a player who intends to cooperate.

$$\mathrm{DC}=3{\left(1-a\right)}^{2}+2a\left(1-a\right)+1\left(1-a\right)a+0a^{2}=3-3a$$

*DC**: expected return for a player who intends to defect this round but apologize for defecting, playing a player who intends to cooperate.

$$DC^{*}=\left(3-c\right){\left(1-a\right)}^{2}+2a\left(1-a\right)+\left(1-c\right)\left(1-a\right)a+0a^{2}=3-3a-c\left(1-a\right)$$

*DD*: expected return for a player who intends to defect this round, playing a player who also intends to defect.

$$\mathrm{DD}=1{\left(1-a\right)}^{2}+0a\left(1-a\right)+3\left(1-a\right)a+2a^{2}=1+a$$

*DD**: expected return for a player who intends to defect this round but apologize for it, playing a player who also intends to defect.

$$DD^{*}=\left(1-c\right){\left(1-a\right)}^{2}+0a\left(1-a\right)+\left(3-c\right)\left(1-a\right)a+2a^{2}=1+a-c\left(1-a\right)$$

#### Total Expected Return

In what follows, *Exp(A, B)* denotes the total return payoff for a player using strategy *A* against a player using strategy *B*. The expected return each round is the sum of the each of the above single-round expected payoffs times the probability that they will be instantiated. When grim trigger players are involved, this probability is based on the error rate *a* for their opponent the previous round, as well as probability *p* of a faker’s apology working in the case of guilt-prone players vs. fakers. The total expected return, then, is the sum of the expected return each round *i*, times the probability *n^i^* that there will be an *i*th round.^1^

$$\begin{array}{llll} \mathrm{Exp}\left(C,C\right) & =\sum_{i=0}^{\infty }\;\mathrm{CC}\left(n^{i}\right)=\left(2-a\right)\sum_{i=0}^{\infty }\;n^{i}=\frac{2-a}{1-n}\; & & \;\\ \mathrm{Exp}\left(C,D\right) & =\mathrm{Exp}\left(C,F\right)=\sum_{i=0}^{\infty }\;\mathrm{CD}\left(n^{i}\right)=3a\sum_{i=0}^{\infty }\;n^{i}=\frac{3a}{1-n}\; & & \;\\ \mathrm{Exp}\left(D,C\right) & =\sum_{i=0}^{\infty }\;\mathrm{DC}\left(n^{i}\right)=\left(3-3a\right)\sum_{i=0}^{\infty }\;n^{i}=\frac{3-3a}{1-n}\; & & \;\\ \mathrm{Exp}\left(D,D\right) & =\mathrm{Exp}\left(D,F\right)=\sum_{i=0}^{\infty }\;\mathrm{DD}\left(n^{i}\right)=\left(1+a\right)\sum_{i=0}^{\infty }\;n^{i}=\frac{1+a}{1-n}\; & & \;\\ \mathrm{Exp}\left(\mathrm{GP},\mathrm{GP}\right) & =\sum_{i=0}^{\infty }\;CC^{*}\left(n^{i}\right)=\left(2-a-\mathrm{ca}\right)\sum_{i=0}^{\infty }\;n^{i}=\frac{2-a-\mathrm{ca}}{1-n}\; & & \;\\ \mathrm{Exp}\left(\mathrm{GP},C\right) & =\sum_{i=0}^{\infty }\;\left[{\left(1-a\right)}^{i}CC^{*}+\left(1-{\left(1-a\right)}^{i}\right)DC^{*}\right]n^{i}\; & & \;\\ & =\sum_{i=0}^{\infty }\;\left[{\left(1-a\right)}^{i}\left(2-a-\mathrm{ca}\right)+\left(1-{\left(1-a\right)}^{i}\right)\left(3-2a-c\right)\right]n^{i}\; & & \;\\ & =\frac{2a^{2}n+\mathrm{ac}-4\mathrm{an}+a+2n-2}{\left(n-1\right)\left(\mathrm{an}-n+1\right)}\; & & \;\\ & \; & \end{array}$$

$$\begin{array}{llll} \mathrm{Exp}\left(\mathrm{GP},D\right) & =\sum_{i=0}^{\infty }\;\left[\left(1-a^{i}\right)DD^{*}+a^{i}CD^{*}\right]n^{i}\; & & \;\\ & =\sum_{i=0}^{\infty }\;\left[\left(1-a^{i}\right)\left(1+a-c\left(1-a\right)\right)+a^{i}\left(3a-\mathrm{ca}\right)\right]n^{i}\; & & \;\\ & =\frac{-a^{2}\mathrm{cn}-a^{2}n+3\mathrm{acn}-\mathrm{ac}-3\mathrm{an}+3a-\mathrm{cn}+n}{\left(n-1\right)\left(\mathrm{an}-1\right)}\; & & \;\\ \mathrm{Exp}\left(F,D\right) & =\mathrm{Exp}\left(F,F\right)=\sum_{i=0}^{\infty }\;DD^{*}\left(n^{i}\right)=\left(1+a-c\left(1-a\right)\right)\sum_{i=0}^{\infty }\;\left(n^{i}\right)\; & & \;\\ & =\frac{1+a-c\left(1-a\right)}{1-n}\; & & \;\\ \mathrm{Exp}\left(F,C\right) & =\sum_{i=0}^{\infty }\;DC^{*}\left(n^{i}\right)=\left(3-2a-c\right)\sum_{i=0}^{\infty }\;n^{i}=\frac{3-2a-c}{1-n}\; & & \;\\ \mathrm{Exp}\left(\mathrm{GT},D\right) & =\mathrm{Exp}\left(\mathrm{GT},F\right)=\sum_{i=0}^{\infty }\;\left[\left(1-a^{i}\right)\mathrm{DD}+a^{i}\mathrm{CD}\right]n^{i}\; & & \;\\ & =\sum_{i=0}^{\infty }\;\left[\left(1-a^{i}\right)\left(1+a\right)+3a^{i+1}\right]n^{i}\; & & \;\\ & =\frac{-a^{2}n-3\mathrm{an}+3a+n}{\left(n-1\right)\left(\mathrm{an}-1\right)}\; & & \;\\ \mathrm{Exp}\left(\mathrm{GT},C\right) & =\sum_{i=0}^{\infty }\;\left[{\left(1-a\right)}^{i}\mathrm{CC}+\left(1-{\left(1-a\right)}^{i}\right)\mathrm{DC}\right]n^{i}\; & & \;\\ & =\sum_{i=0}^{\infty }\;\left[{\left(1-a\right)}^{i}\left(2-a\right)+\left(1-{\left(1-a\right)}^{i}\right)\left(3-3a\right)\right]n^{i}\; & & \;\\ & =\frac{3a^{2}n-4\mathrm{an}+a+2n-2}{\left(n-1\right)\left(\mathrm{an}-n+1\right)}\; & & \; \end{array}$$

$$\begin{array}{llll} \mathrm{Exp}\left(F,\mathrm{GP}\right) & =\sum_{i=0}^{\infty }\;\left[{\left(p-\mathrm{ap}+a\right)}^{i}DC^{*}+\left(1-{\left(p-\mathrm{ap}+a\right)}^{i}\right)DD^{*}\right]n^{i}\; & & \;\\ & =\sum_{i=0}^{\infty }\;\left[{\left(p-\mathrm{ap}+a\right)}^{i}\left(3-3a-c+\mathrm{ca}\right)+\left(1-{\left(p-\mathrm{ap}+a\right)}^{i}\right)\left(1+a-c+\mathrm{ca}\right)\right]n^{i}\; & & \;\\ & =\frac{\left(a-1\right)\left(c\left(\mathrm{an}\left(p-1\right)-\mathrm{np}+1\right)+n\left(a\left(p-1\right)+p+2\right)-3\right)}{\left(1-n\right)\left(\mathrm{an}\left(p-1\right)-\mathrm{np}+1\right)}\; & & \;\\ \mathrm{Exp}\left(\mathrm{GP},F\right) & =\sum_{i=0}^{\infty }\;\left[{\left(p-\mathrm{ap}+a\right)}^{i}CD^{*}+\left(1-{\left(p-\mathrm{ap}+a\right)}^{i}\right)DD^{*}\right]n^{i}\; & & \;\\ & =\sum_{i=0}^{\infty }\;\left[{\left(p-\mathrm{ap}+a\right)}^{i}\left(3a-\mathrm{ca}\right)+\left(1-{\left(p-\mathrm{ap}+a\right)}^{i}\right)\left(1+a-c+\mathrm{ca}\right)\right]n^{i}\; & & \;\\ & =\frac{-a^{2}\mathrm{cnp}+a^{2}\mathrm{cn}-a^{2}\mathrm{np}+a^{2}n+2\mathrm{acnp}-3\mathrm{acn}+\mathrm{ac}+3\mathrm{an}-3a-\mathrm{cnp}+\mathrm{cn}+\mathrm{np}-n}{\left(n-1\right)\left(\mathrm{anp}-\mathrm{an}-\mathrm{np}+1\right)}\; & & \;\\ \mathrm{Exp}\left(C,\mathrm{GP}\right) & =\mathrm{Exp}\left(C,\mathrm{GT}\right)=\sum_{i=0}^{\infty }\;\left[{\left(1-a\right)}^{i}\mathrm{CC}+\left(1-{\left(1-a\right)}^{i}\right)\mathrm{CD}\right]n^{i}\; & & \;\\ & =\sum_{i=0}^{\infty }\;\left[{\left(1-a\right)}^{i}\left(2-a\right)+\left(1-{\left(1-a\right)}^{i}\right)\left(3a\right)\right]n^{i}\; & & \;\\ & =\frac{-3a^{2}n-\mathrm{an}+a+2n-2}{\left(n-1\right)\left(\mathrm{an}-n+1\right)}\; & & \;\\ \mathrm{Exp}\left(D,\mathrm{GP}\right) & =\mathrm{Exp}\left(D,\mathrm{GT}\right)=\sum_{i=0}^{\infty }\;\left[\left(1-a^{i}\right)\mathrm{DD}+a^{i}\mathrm{DC}\right]n^{i}\; & & \;\\ & =\sum_{i=0}^{\infty }\;\left[\left(1-a^{i}\right)\left(1+a\right)+a^{i}\left(3-3a\right)\right]n^{i}\; & & \;\\ & =\frac{\left(a-1\right)\left(\left(a-2\right)n+3\right)}{\left(1-n\right)\left(\mathrm{an}-1\right)}\; & & \;\\ \mathrm{Exp}\left(F,\mathrm{GT}\right) & =\sum_{i=0}^{\infty }\;\left[a^{i}DC^{*}+\left(1-a^{i}\right)DD^{*}\right]n^{i}\; & & \;\\ & =\sum_{i=0}^{\infty }\;\left[a^{i}\left(3-2a-c\right)+\left(1-a^{i}\right)\left(1+a-c\left(1-a\right)\right)\right]n^{i}\; & & \;\\ & =\frac{-a^{2}\mathrm{cn}-a^{2}n+2\mathrm{acn}+2\mathrm{an}-2a-c-2n+3}{\left(n-1\right)\left(\mathrm{an}-1\right)}\; & & \; \end{array}$$

*Exp*(*GT, GT*), *Exp*(*GT, GP*), and *Exp*(*GP, GT*) are a bit more complicated. Luckily, though, the first two are equal to one another and the third follows analogously. I computed *Exp*(*GT, GT*) as follows:

Note that the expected return for round 1 in this case is:

$$E_{1}=\mathrm{CC}$$

Next, note that if we write the expectation for each round *i* as

$$E_{i}=x\left(i\right)\mathrm{CC}+y\left(i\right)\mathrm{CD}+z\left(i\right)\mathrm{DC}+w\left(i\right)\mathrm{DD},$$

we get that

$$\begin{array}{llll} E_{i+1} & ={\left(1-a\right)}^{2}x\left(i\right)\mathrm{CC}+\left(a\left(1-a\right)x\left(i\right)+\mathrm{ay}\left(i\right)\right)\mathrm{CD}\; & & \;\\ & \;+\left(a\left(1-a\right)x\left(i\right)+\mathrm{az}\left(i\right)\right)\mathrm{DC}+\left(a^{2}x\left(i\right)+\left(1-a\right)y\left(i\right)+\left(1-a\right)z\left(i\right)+w\left(i\right)\right)\mathrm{DD}.\; & & \; \end{array}$$

Considering *x, y, z*, and *w* as recursive functions with initial values *x*(0) = 1, *y*(0) = *z*(0) = *w*(0) = 0 (and plugging into Mathematica), we get:

$$\begin{array}{llll} x\left(i\right) & ={\left(1-a\right)}^{2i-2}\; & & \;\\ y\left(i\right) & =z\left(i\right)=\frac{a(a^{i}-{\left(1-a\right)}^{2i}}{\left(a-1\right)\left(\left(a-3\right)a+1\right)}\; & & \;\\ w\left(i\right) & =\frac{a\left(-2\left(a-1\right)a^{i}+a{\left(1-a\right)}^{2i}+{\left(1-a\right)}^{2i}+a-3\right)-{\left(1-a\right)}^{2i}+1}{{\left(a-1\right)}^{2}\left(\left(a-3\right)a+1\right)}\; & & \; \end{array}$$

And at last we can express our final three expectations:

$$\begin{array}{llll} \mathrm{Exp}\left(\mathrm{GT},\mathrm{GT}\right) & =\mathrm{Exp}\left(\mathrm{GT},\mathrm{GP}\right)\; & & \;\\ & =\sum_{i=0}^{\infty }\left[{\left(1-a\right)}^{2i-2}\mathrm{CC}+\frac{a\left(a^{i}-{\left(1-a\right)}^{2i}\right)}{\left(a-1\right)\left(\left(a-3\right)a+1\right)}\left(\mathrm{CD}+\mathrm{DC}\right)\right.\; & & \;\\ & \left.\;+\frac{a\left(-2\left(a-1\right)a^{i}+a{\left(1-a\right)}^{2i}+{\left(1-a\right)}^{2i}+a-3\right)-{\left(1-a\right)}^{2i}+1}{{\left(a-1\right)}^{2}\left(\left(a-3\right)a+1\right)}\mathrm{DD}\right]n^{i}\; & & \;\\ & =\sum_{i=0}^{\infty }\left[{\left(1-a\right)}^{2i-2}\left(2-a\right)+\frac{a\left(a^{i}-{\left(1-a\right)}^{2i}\right)}{\left(a-1\right)\left(\left(a-3\right)a+1\right)}\left(3\right)\right.\; & & \;\\ & \left.\;+\frac{a\left(-2\left(a-1\right)a^{i}+a{\left(1-a\right)}^{2i}+{\left(1-a\right)}^{2i}+a-3\right)-{\left(1-a\right)}^{2i}+1}{{\left(a-1\right)}^{2}\left(\left(a-3\right)a+1\right)}\left(1+a\right)\right]n^{i}\; & & \;\\ & =\frac{-2+a-\left(-1+a\right)\left(2+a^{2}\right)n-a\left(1+\left(-3+a\right)a^{2}\right)n^{2}}{{\left(-1+a\right)}^{2}\left(-1+n\right)\left(-1+{\left(-1+a\right)}^{2}n\right)\left(-1+\mathrm{an}\right)}\; & & \; \end{array}$$

$$\begin{array}{llll} \mathrm{Exp}\left(\mathrm{GP},\mathrm{GT}\right) & =\sum_{i=0}^{\infty }\left[{\left(1-a\right)}^{2i-2}CC^{*}+\frac{a\left(a^{i}-{\left(1-a\right)}^{2i}\right)}{\left(a-1\right)\left(\left(a-3\right)a+1\right)}\left(CD^{*}+DC^{*}\right)\right.\; & & \;\\ & \;\left.+\frac{a\left(-2\left(a-1\right)a^{i}+a{\left(1-a\right)}^{2i}+{\left(1-a\right)}^{2i}+a-3\right)-{\left(1-a\right)}^{2i}+1}{{\left(a-1\right)}^{2}\left(\left(a-3\right)a+1\right)}DD^{*}\right]n^{i}\; & & \;\\ & =\sum_{i=0}^{\infty }\left[{\left(1-a\right)}^{2i-2}\left(2-a-\mathrm{ca}\right)+\frac{a\left(a^{i}-{\left(1-a\right)}^{2i}\right)}{\left(a-1\right)\left(\left(a-3\right)a+1\right)}\left(3-c\right)\right.\; & & \;\\ & \;\left.+\frac{a\left(-2\left(a-1\right)a^{i}+a{\left(1-a\right)}^{2i}+{\left(1-a\right)}^{2i}+a-3\right)-{\left(1-a\right)}^{2i}+1}{{\left(a-1\right)}^{2}\left(\left(a-3\right)a+1\right)}\left(1+a-c\left(1-a\right)\right)\right]n^{i}\; & & \;\\ & =\left[-\left(a^{4}cn^{2}\right)+5a^{3}cn^{2}-4a^{2}cn^{2}+\mathrm{ac}n^{2}-a^{4}n^{2}+3a^{3}n^{2}-an^{2}-a^{3}\mathrm{cn}-a^{2}\mathrm{cn}-a^{3}n+a^{2}n\right.\; & & \\ & \;\left.\;-2\mathrm{an}+2n+\mathrm{ac}+a-2\right]/\left[{\left(-1+a\right)}^{2}\left(-1+n\right)\left(-1+\mathrm{an}\right)\left(-1+n-2\mathrm{an}+a^{2}n\right)\right]\; & & \; \end{array}$$

^1^The more complicated of these infinite sums are calculated in Mathematica.
